# Insulin induces a positive relationship between the rates of ATP and glycogen changes in isolated rat liver in presence of glucose; a ^31^P and ^13^C NMR study

**DOI:** 10.1186/1743-7075-2-32

**Published:** 2005-11-21

**Authors:** Laurence Baillet-Blanco, Marie-Christine Beauvieux, Henri Gin, Vincent Rigalleau, Jean-Louis Gallis

**Affiliations:** 1 Service de Diabétologie-Nutrition, Hôpital du Haut-Lévêque, Avenue de Magellan, F-33604 Pessac, France; 2 Centre de Résonance Magnétique des Systèmes Biologiques, UMR 5536 CNRS-Université Bordeaux 2, 146 rue Léo Saignat, F-33076 Bordeaux Cedex, France

**Keywords:** insulin, ATP, glycogen, oxidative phosphorylation, liver

## Abstract

**Background:**

There is an emerging theory suggesting that insulin, which is known to be the predominant postprandial anabolic hormone, is also a major regulator of mitochondrial oxidative phosphorylation in human skeletal muscle. However, little is known about its effects in the liver. Since there is a theoretical relationship between glycogen metabolism and energy status, a simultaneous and continuous investigation of hepatic ATP and glycogen content was performed in intact and isolated perfused liver by ^31^P and ^13^C nuclear magnetic resonance (NMR) The hepatic rates of ATP and glycogen changes were evaluated with different concentrations of insulin and glucose during continuous and short-term supply.

**Results:**

Liver from rats fed *ad libitum *were perfused with Krebs-Henseleit Buffer (KHB)(controls) or KHB containing 6 mM glucose, 30 mM glucose, insulin alone, insulin + 6 mM glucose, insulin + 30 mM glucose. In the control, glycogenolysis occurred at a rate of -0.53 ± 0.021 %·min^-1^ and ATP content decreased at a rate of -0.28 ± 0.029 %·min^-1^. In the absence of insulin, there was a close proportional relationship between the glycogen flux and the glucose concentration, whereas ATP rates never varied. With insulin + glucose, both glycogen and ATP rates were strongly related to the glucose concentration; the magnitude of net glycogen flux was linearly correlated to the magnitude of net ATP flux: flux_glycogen _= 72.543(flux_ATP_) + 172.08, R^2 ^= 0.98.

**Conclusion:**

Only the co-infusion of 30 mM glucose and insulin led to (i) a net glycogen synthesis, (ii) the maintenance of the hepatic ATP content, and a strong positive correlation between their net fluxes. This has never previously been reported. The specific effect of insulin on ATP change is likely related to a rapid stimulation of the hepatic mitochondrial oxidative phosphorylation. We propose that variations in the correlation between rates of ATP and glycogen changes could be a probe for insulin resistance due to the action of substrates, drugs or pathologic situations. Consequently, any work evaluating insulin resistance on isolated organs or *in vivo* should determine both ATP and glycogen fluxes.

## Background

In the metformin treatment of insulin resistance-related complications, the mitochondrial effects of the drug are probably crucial in explaining its unique efficacy [1]. Mitochondrial dysfunctions have been reported in the muscle in type 2 diabetes [2] and in age-related insulin resistance [3], suggesting a link between insulin action and oxidative capacity in humans [4]. Thus, in healthy humans, it has been demonstrated that high physiological insulin sustained stimulated muscle protein synthesis and mitochondrial ATP production rate for 8 hr [5]. However, a rapid stimulatory action of insulin on ATP production was not shown.

Owing to its strong capacity for glucose production and utilization, the liver is a key regulator of glucose homeostasis. One of its major functions is to store glucose as glycogen after meals (glycogen synthesis) and to release glucose from this glycogen (glycogenolysis) at the post-absorptive state, which accounts for most endogenous glucose production. Disturbance of this function is thought to play a major role in the hyperglycemia of type 2 diabetes and in other insulin-resistant states. Despite much work on the issue, the effect of insulin on hepatic glycogenosynthesis remains controversial: insulin is known to activate glycogen synthase *in vitro *[6], but hepatic glycogenesis *in vivo *seems to need an increase in both insulin and plasma glucose levels.

Glucose is the main energy substrate and its hepatic metabolism can lead to ATP production during glycogenolysis (by cytosolic glycolysis and mitochondrial oxidative phosphorylation) or direct ATP consumption during glycogen synthesis (or indirectly from gluconeogenesis). Despite this strong link, no study to our knowledge has simultaneously addressed the effects of insulin and glucose on the rates of changes of hepatic glycogen and ATP contents. This knowledge gap is particularly regrettable as some studies have reported abnormal hepatic ATP contents in insulin-resistant states as obesity [7] and nonalcoholic steatohepatitis [8].

Nuclear magnetic resonance spectroscopy (NMR) is the only technique that allows non-invasive repetitive and simultaneous quantitation of both glycogen and ATP contents in the isolated liver. Monitoring in real-time makes it possible to calculate the rates of change in metabolites. We chose to work on isolated whole liver, because it is not subject to hormonal or nutritional parameters (in contrast to *in vivo *models). Moreover, the isolated whole liver is a closer model to physiological conditions than isolated hepatocytes. The initial presence of hepatic glycogen is the prerequisite for the kinetic study of its rate of change. As the glycogen level is very low in the fasting state, this study was performed on isolated livers from fed rats.

The purpose of this work was to explore in the liver the relationship between the glycogen pathway and energy metabolism and their dependence on insulin and/or glucose supply.

## Results

### (1) Rates of change of liver glycogen content (Table 1)

**Table 1:** Rates of hepatic glycogen changes and ATP changes (nmol·min^-1^·g^-1 ^of liver ww).

	**Experimental conditions**					
	**Control (KHB) ** Group A, n = 7	**G 6 mM ** Group B, n = 8	**G 30 mM ** Group C, n = 8	**Insulin ** Group D, n = 5	**Insulin + G 6 mM ** Group E, n = 5	**Insulin + G 30 mM ** Group F, n = 5

**GLYCOGEN**	**-387 ± 73**	(a) -64 ± 109* *p = 0.02 vs control* **(b) -474 ± 102 ** *NS vs control*	(a) +52 ± 95** *p = 0.003 vs control* **(b) -277 ± 80 ** *NS vs control*	**-431 ± 29 ** *NS vs control*	**-29 ± 73 ** *p < 0.003 vs insulin NS vs G6(a)*	**+139 ± 47 ** *p < 0.00001 vs insulin* *p = 0.015 vs G30(a)*
**ATP**	**-7.28 ± 0.76**	**-10.14 ± 1.61 ** *NS vs control*	**-5.98 ± 1.22 ** *NS vs control*	**-8.06 ± 1.22 ** *NS vs control*	**-3.38 ± 0.17 ** *p = 0.03 vs insulin* *p = 0.01 vs G6*	**-0.10 ± 0.42 ** *p = 0.003 vs insulin* *p = 0.006 vs G30*

100% glycogen = 73 ± 8.5 μmol glycosyl units·g^-1 ^of liver ww; 100% ATP = 2.60 ± 0.65 μmol·g^-1 ^of liver ww. The rates of glycogen changes were biphasic in group B and C; the first phase (a) lasted *20 min or **60 min. Mean ± SEM.

**Figure 1:**
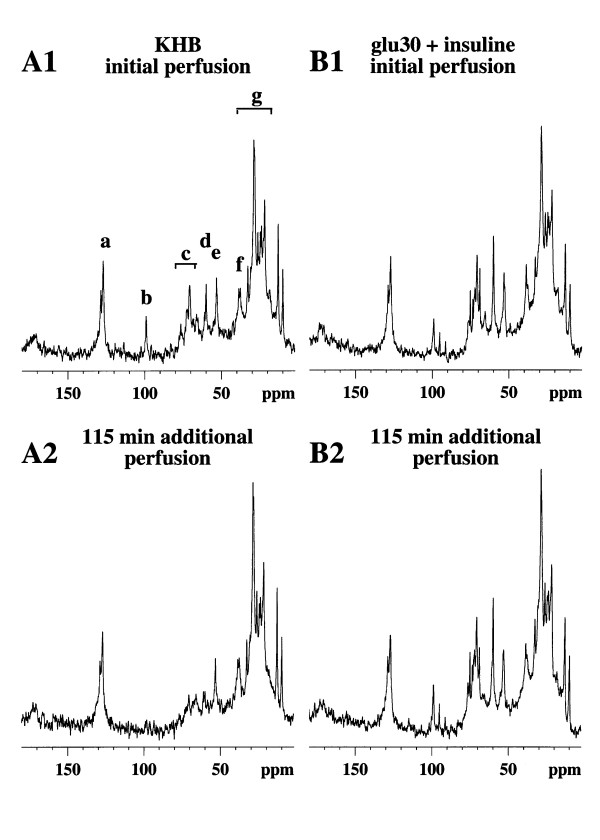
**Natural abundance ^13^C NMR spectra of two typical experiments at the beginning (A1 and B1) and after 115 min of perfusion (A2 and B2)**. A1 and A2 were ^13^C NMR spectra from a control rat perfused with Krebs-Henseleit buffer alone and B1 and B2 were ^13^C NMR spectra from rats perfused with both 30 mM glucose and insulin. Major resonances are assigned to (a,g) fatty acid, (b) C-1 glycogen, (c) glucose and glycogen (C-3β, C-5β glucose, glycogen; C-2 glucose, C-3α glucose; C2, C-5α glucose, C-5 glycogen; C-4αβ glucose, glycogen), (d) C-6 glucose, glycogen, (e) choline, (f) ethanolamine. Note the C-1 glycogen (b) decrease in the KHB group (A1 and A2) and the relative stability of the glycogen content of the liver during perfusion of both 30 mM glucose and insulin (B1 and B2).

Since glycogen has been shown to be nearly 100% NMR-visible [9], changes in the peak area of the C1 resonance of glycogen can be used to monitor and calculate the rate of change in glycogen content in real time. In the absence of glucose in the isotonic perfusion medium (KHB, control group A), a linear decrease in glycogen content occurred without any lag time and its rate of disappearance was -0.53 ± 0.021 %·min^-1^ (100% being considered as the initial content = 73 ± 8.5 μmol glycosyl units·g^-1 ^liver wet weight) (fig. 1).

**Figure 2:**
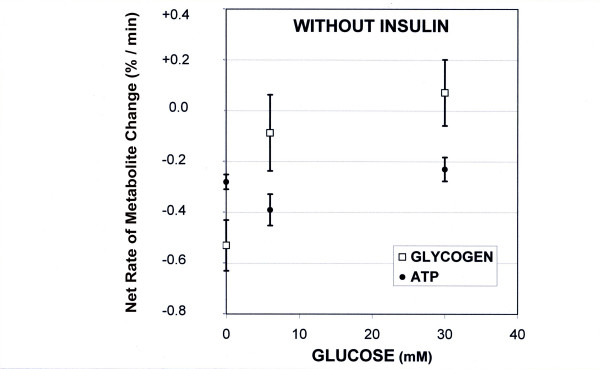
**Rates of liver glycogen and ATP changes in absence of insulin in the perfusate**. The results are expressed in %·min^-1^ (mean ± SEM; 100% initial glycogen content = 73 ± 8.5 μmol glycosyl units·g^-1 ^liver wet weight; 100% initial ATP content = 2.60 ± 0.65 μmol/g liver wet weight). Only the rate of glycogen content increased with the perfusate glucose concentration.

In the presence of glucose in the KHB medium (Group B and C), the glycogen content tended to remain near the initial level for 20 min (6 mM glucose) and for 50 min with 30 mM glucose. During this step, a slight decrease in glycogen content with a rate of -0.087 ± 0.29 %·min^-1 ^(p = 0.022 vs. control) occurred in the 6 mM glucose group and an increase in glycogen content was observed (+0.071 ± 0.13%·min^-1^, n = 4, p = 0.002 vs. KHB perfusion) in the 30 mM glucose group (fig 2). This positive balance between glycogenolysis and glycogenosynthesis was about 52 nmoles glucosyl units·min^-1^·g^-1^. After this relatively stable initial period, the apparent rate of glycogenolysis was similar in all the groups (-0.65 ± 0.14 and -0.38 ± 0.11%·min^-1^, in 6 mM and 30 mM glucose groups respectively; NS vs. KHB perfusion).

**Figure 3:**
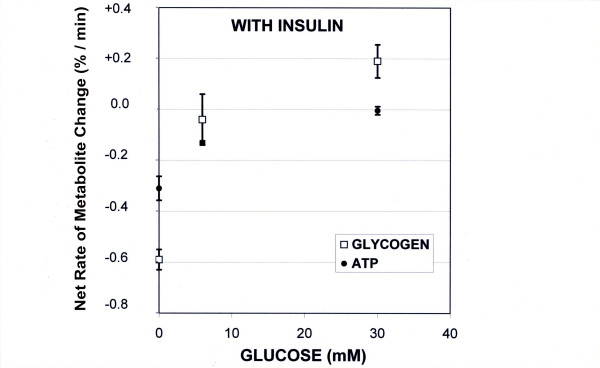
**Rates of liver glycogen and ATP changes in presence of insulin in the perfusate**. The results are expressed in %·min^-1 ^(mean ± SEM; 100% initial glycogen content = 73 ± 8.5 μmol glycosyl units·g^-1^ liver wet weight; 100% initial ATP content = 2.60 ± 0.65 μmol/g liver wet weight). The rates of both ATP and glycogen contents increased with the perfusate glucose concentration.

With insulin alone in the KHB at different concentrations (Group D) (60 mU/L, 120 mU/L or 600 mU/L), the decrease in glycogen content was similar to that in the KHB group. The results are expressed with different insulin doses in the same group D for purposes of comparison with the other liver groups (-0.59 ± 0.04%·min^-1^, n = 9) (fig. 3)

**Figure 4:**
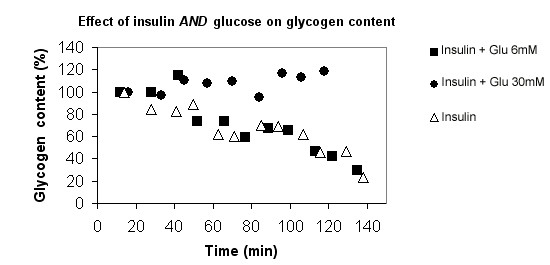
**Time course of the change in hepatic glycogen content throughout the entire protocol perfusion in presence of insulin**. The results are presented for three typical individual experiments: insulin alone (group D), insulin + 6 mM glucose (group E) and insulin + 30 mM glucose (group F). The results are expressed as the percentage of initial glycogen content, 100% being the value at the onset of the experiment. Note that the decrease in glycogen content observed with insulin alone was similar to that in the KHB control group.

In the presence of both 6 mM glucose and insulin (Group E) the initial liver glycogen content was maintained for a period ranging from 45 to 90 min (slight decrease in glycogen content of -0.04 ± 0.38%·min^-1^, p = 0.01 vs. insulin group (group D) and NS vs. 6 mM glucose alone (group B). In the presence of both 30 mM glucose and insulin in the medium (group F), we observed throughout perfusion an increase in the rate of glycogen change in the isolated liver (+0.19 ± 0.065%·min^-1^, p = 0.0001 vs. KHB perfusion; p < 0.0001 vs. insulin alone; NS vs. 30 mM glucose alone) (fig. 3 and 4), leading to a glycogen content 2.6-fold higher than in glucose 30 mM. This positive balance was about 139 nmoles glycosyl units·min^-1^·g^-1^.

### (2) Rates of Change of liver ATP content (Table 1)

**Figure 5:**
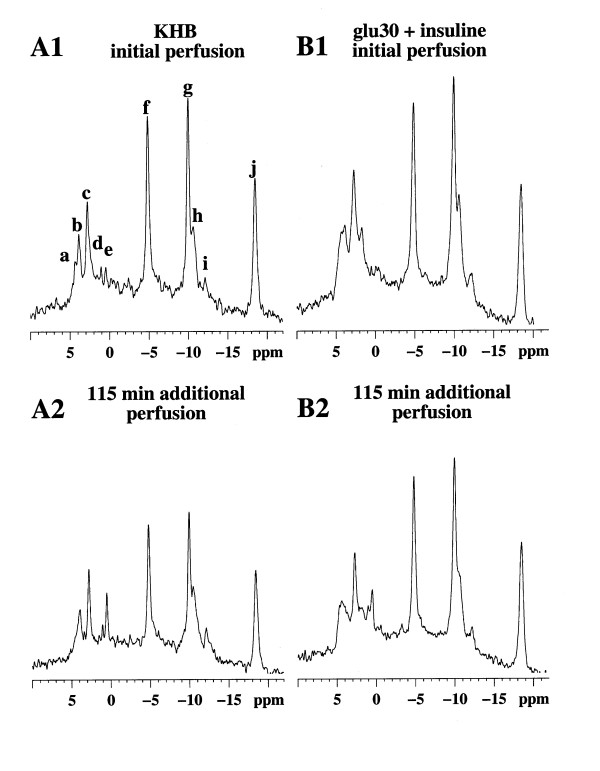
**^31^P NMR spectra of 2 typical experiments at the beginning (A1 and B1) and after 115 min of perfusion (A2 and B2)**. A1 and A2 were ^31^P NMR spectra from a control rat perfused with Krebs-Henseleit buffer alone and B1 and B2 were ^31^P NMR spectra from rat perfused with both 30 mM glucose and insulin. Major resonances are assigned to (a) phosphomonoesters, (b) phosphocholine, (c) intracellular inorganic phosphate, (d) glycerol-3-phosphorylcholine, (f) nucleoside-5'-triphosphates (γNTP) and diphosphates (βNDP), (g) α-NTP and α-NDP, (h,i) nicotinamide adenine dinucleotide and uridine-5'-diphosphoglucose, (j) βNTP. The external reference is not shown (18.40 ppm). Note the βATP (j) decrease in the KHB group (A1 and A2) and the relative stability of the ATP content of the liver during the perfusion of both 30 mM glucose and insulin (B1 and B2).

In the absence of insulin, the liver ATP content in the control group (0 glucose) decreased at a rate of -0.28 ± 0.029 %·min^-1^, near -7.3 nmol. min^-1^·g^-1^, 100% ATP corresponding to 2.60 ± 0.65 μmol/g liver wet weight (fig. 2 and 5). Similar rates were observed with 6 mM glucose (group B; -0.39 ± 0.062) or with 30 mM glucose (group C; -0.23 ± 0.047 %·min^-1^) (fig 2).

In the presence of insulin alone in the medium (group D), there was also no significant difference compared to the control group. At all insulin concentrations, no difference was observed in ATP evolution so the results are pooled in group D (-0.31 ± 0.047%·min^-1^, n = 9).

**Figure 6:**
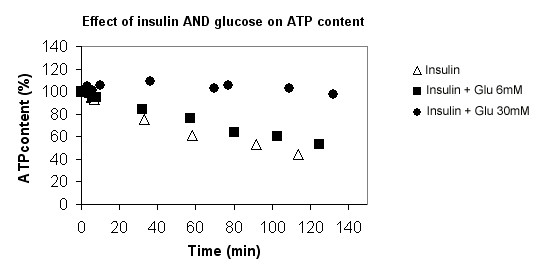
**Time course of the change in hepatic ATP content throughout the entire protocol perfusion in presence of insulin**. The results are presented for three typical individual experiments: insulin alone (group D), insulin + 6 mM glucose (group E) and insulin + 30 mM glucose (group F). The results are expressed as the percentage of initial ATP content, 100% being the value at the onset of the experiment. Note that in the control KHB group, the 6 mM glucose group and the 30 mM glucose group, the ATP content decreased at a similar rate to the insulin group.

In the presence of both insulin and glucose (group E and F), two different responses were observed: (i) with 6 mM glucose (group E) a decrease in the ATP content (-0.13 ± 0.006 %·min^-1^) similar to that in the control group (ii) with 30 mM glucose (group F) the rate of ATP decrease was almost zero; -0.004 ± 0.016 %·min^-1^) (fig 3), so 100% of the initial content was maintained during the first 90 min (fig. 6). This latter rate was significantly lower than with 30 mM glucose alone (p = 0.031) or insulin alone (p < 0.001).

### (3) Relationship between rates of hepatic glycogen and ATP changes

**Figure 7:**
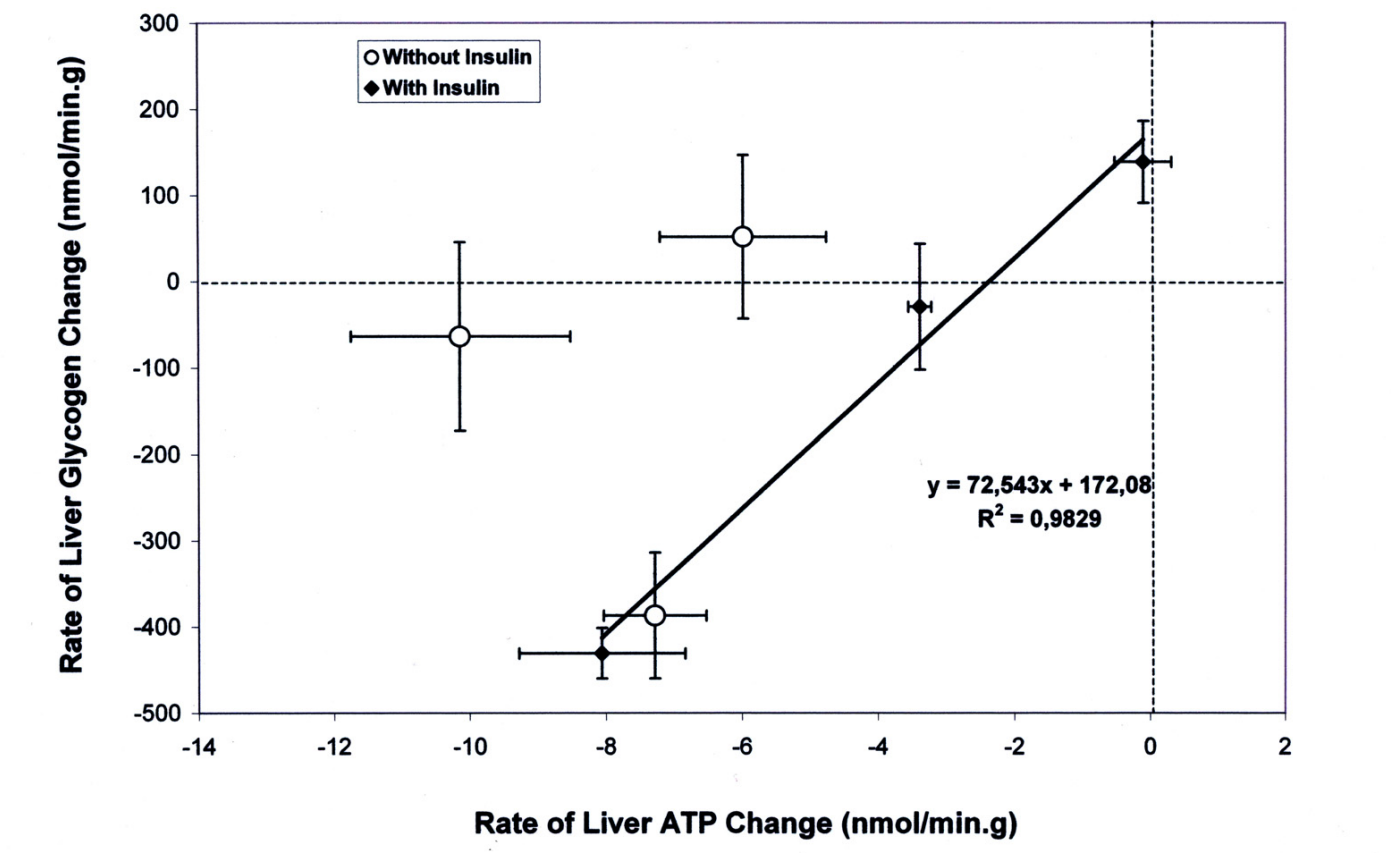
**Correlation between the rates of ATP and glycogen changes in presence of insulin according to the perfusate glucose concentration**. The rates are expressed in nmol·min^-1^·g^-1^. The equation for the linear correlation is flux_glycogen _= 72.543(flux_ATP_) + 172.08, R^2 ^= 0.98. To obtain a positive net flux of glycogen (net synthesis), the net ATP consumption must not exceed 2.4 nmol·min^-1^·g^-1^.

Simultaneous analysis of ATP and glycogen contents under the same experimental conditions showed differences in the evolution of the net metabolic flux that was closely dependent on the presence or absence of insulin. In the absence of insulin, there was a close proportional relationship between the glycogen flux and the concentration of glucose supply, whereas the ATP rates were independent of the glucose concentration (fig 2). Conversely, in the presence of both glucose and insulin, glycogen and ATP rates were both strongly related to the perfusate glucose concentration (fig 3). The magnitude of net glycogen flux was thus correlated with the net ATP flux : flux_glycogen _= 72.543(flux_ATP_) + 172.08, R^2 ^= 0.98 (fig 7), and the final glycogen content was significantly higher than in the presence of glucose alone.

## Discussion

Recent evidence supports the hypothesis that insulin, the key postprandial hormone involved in fuel metabolism, is also a major regulator of mitochondrial oxidative phosphorylation in human skeletal muscle [4,5]. However, little is known about its effects in the liver.

Given the theoretical relationship between glycogen metabolism and energy status, hepatic ATP and glycogen content were investigated in rat liver. Since methods based on extraction of glycogen and/or ATP from liver cannot monitor their evolution in real time in the same intact liver, NMR spectroscopy was used to study the appearance or disappearance of metabolites in the same organ. This work is the first to investigate in real time the rapid effects of glucose and/or insulin on the rates of changes in glycogen and ATP content in the isolated liver of fed rats.

Insulin alone had no effect on the natural decrease in hepatic glycogen and ATP contents during the 2-hr perfusion. The increase in the concentration from 0 to 30 mM of glucose alone in the perfusate maintained liver glycogen in a dose-dependent manner. Moreover, glucose had a glycogen-sparing effect, in agreement with other data [10,11], but had no effect on basal ATP consumption, particularly at 30 mM. Indeed it is known that the glucose concentration must be around 20–30 mM to induce both a decrease in the activity of phosphorylase *a *below 10% (of total activity) and a subsequent activation of the glycogen synthase [6,12].

Only co-perfusion with insulin and 30 mM glucose led to a glycogen synthesis 2.6-fold (260%) higher than with 30 mM glucose alone, and this positive balance was in agreement with previous results obtained in fasted rats in conditions of neoglucogenesis (addition of alanine and/or lactate) [9] or with fructose [11]. This isolated liver model clearly demonstrates that insulin acts directly on the cells, thereby invalidating the hypothesis of an insensitivity of the liver to insulin developed elsewhere [13]. Since glycogen synthesis implies some UTP (and ATP) use, one might expect an increase in ATP consumption resulting in a reduction of liver ATP content. In fact, the presence of both 30 mM glucose and insulin led to (i) the maintenance of the hepatic ATP content, demonstrating that ATP consumption equaled ATP synthesis, and (ii) a net glycogen synthesis reflecting a positive balance between glycogenolysis and glycogen synthesis. More significant is the evidence of a strong linear correlation between the net fluxes of these two metabolites. We did not differentiate between the reduction of glycogenolysis and the enhancement of glycogenosis, or between the separate rates of ATP production and utilization that led to modifying hepatic glycogen and ATP contents. However, our global kinetic study of the balance between the synthesis and lysis pathways clearly underlines for the first time that it is only in the presence of insulin that the net flux of ATP correlates with the net flux of hepatic glycogen synthesis from exogenous glucose in the isolated liver.

To date, the role of insulin in the correlation of the net hepatic flux of ATP and glycogen has not been described, although previous reports point to a link between carbohydrate metabolism and ATP pathways. Insulin and glucose perfusions during resuscitation of rats from hemorrhagic shock increase the hepatic ATP content [14]. Both liver glycogen and ATP contents are decreased by fasting and exercise [15]. Cortez-Pinto *et al *[8] reported that recovery from hepatic ATP depletion was reduced with a body mass increase in healthy humans and was severely impaired in subjects with nonalcoholic steatohepatitis, a condition associated with hepatic insulin resistance. This result is corroborated by an inverse correlation observed between hepatic ATP content and Body Mass Index [7].

In 48 hr-cultured hepatocytes in the presence of insulin, a previous study showed an increase in the ATP concentration from 12 hr with a concomitant close correlation between the increases in exogenous glucose transport rate and glycogen content [16]. However, the authors underlined that no clear effect of insulin on glucose transport was found in the isolated 2 hr-perfused liver system. Moreover, the main role of insulin in maintaining the metabolic competence of cultured hepatocytes could be linked to the absolute ATP concentration rather than to the energy charge [17]. In rat hepatocytes and muscle, insulin, which otherwise has no significant effect on respiration, acts specifically on the mitochondrial Krebs cycle, within 30 sec, to stimulate by 30% the oxidation of only carbons 2 and 3 of pyruvate (or acetate) to CO_2 _[18]. Other authors [19] showed in the isolated hepatic mitochondria of rats treated with insulin for 9 weeks that the hormone improved the function of oxidative phosphorylation by increasing (i) ATP synthase activity, (ii) the ADP/O ratio and (iii) the respiratory control ratio. It may therefore be hypothesized that the rapid action of insulin (in the presence of exogenous glucose) on liver glycogen flux is exerted through the increase in ATP turnover, *via *(i) an enhancement in glycolytic ATP production and/or (ii) an increase in ATP flux originating from oxidative phosphorylation related to the activation of Krebs cycle and/or (iii) an increase in the oxidative phosphorylation yield.

## Conclusion

Beside extending numerous NMR studies using non-physiological conditions (such as clamps to maintain insulin and glucose levels; review in [20]) or expensive ^13^C-labeling [21], this kinetic study performed by natural abundance NMR in isolated liver describes a simple reliable method to analyze the link between ATP and glycogen.

The main finding was a close metabolic link between hepatic ATP and glycogen induced by insulin in the presence of glucose. Such a major role for energy metabolism is in agreement with the emergent hypothesis suggesting a defect in mitochondrial oxidative phosphorylation in insulin-resistance [22]. We propose that variations in the correlation between rates of ATP and glycogen changes could be a probe for insulin resistance action of substrates, drugs or pathologic situations. Using NMR surface coil techniques, it should be possible to address this question in a similar fashion *in vivo *in the rat and in clinical investigations in human. Any work evaluating insulin resistance on isolated organs or *in vivo *should determine both ATP and glycogen fluxes.

## Methods

### Animal perfusion conditions

Male Wistar rats weighing between 80 and 120 g were used. Rats were obtained from the existing colony in the animal unit of Bordeaux 2 University. They were maintained on standard rat chow and were housed in an environmentally controlled room (temperature, humidity and airflow conditions) with a 12-hour light/dark cycle. The standard non-purified diet contained by weight (g/100 g): 63 starch (corn, sorghum, wheat, oats, barley), 22 protein, 3.5 fat, 6 fiber, 1 vitamin mixture and 4 salt mixture (Ralston, Purina, St. Louis, MO). The rats were fed *ad libitum *to facilitate glycogen synthesis and visualization of glycogen liver store at the beginning of the experiment. The rats were anesthetized with intraperitoneal barbiturate injection (pentobarbital sodium: 50 mg/kg of body weight). The rat liver was commonly used to investigate energetic metabolism [23] and glycogen turn-over [9]. The liver (4–6 g) from rats was perfused *in situ *through the portal vein (anterograde perfusion technique) as previously described [23]. The bile duct was cannulated in order to avoid mixing of bile with the surrounding medium. The perfusion was performed at 37°C with 95% O_2_–5% CO_2_ gassed Krebs-Henseleit buffer (KHB) as a flow rate (5 ml/min·g wet weight (ww)). The KHB composition was (mmol/L): 120 NaCl, 4.70 KCl, 1.20 MgSO_4_, 25 NaHCO_3_, 1.20 K_2_HPO_4_-KH_2_PO_4_, 1.30 CaCl_2_, 0.3 mM Na-pyruvate and 2.10 mM Na-lactate (pH = 7.35 at 37°C). The temperature of the perfusion circuit was regulated with a thermostatic bath. The liver (perfused in a non-recirculating mode) was then excised from the rat abdomen and transferred to a 20-mm diameter NMR cell. The perfusate temperature and pH were monitored before entering and after leaving the liver by continuous flow pH electrodes and temperature probes. High-grade chemicals were purchased from Sigma Chemicals (Saint Louis MO) except where otherwise specified.

This study complied with NIH guidelines (national research 1985).

### Experimental procedures

Different groups of liver were perfused for 150 min under various medium concentrations of glucose and insulin (Actrapid, Novo Nordisk A/S 2880 Bagsvaerd Danemark). Insulin and glucose concentrations in this work reflect the portal concentration in *in vivo *conditions [24-26]. 60 mU/L of insulin is known to be the physiological level in the portal vein in the fasting state but this concentration probably increases in the fed state [24]. To study the effect of insulin on the liver, we chose to use (i) the physiological concentration, and (ii) 2- and 10-fold this concentration to mimic the fed portal state. Plasma glucose concentration in rats is around 6 mmol/L but the level in the portal vein during the fed state is higher (around 30 mmol/L) [25,26].

- Group A: control group (KHB alone) (n = 7)

- Group B: D-glucose 6 mM (n = 8)

- Group C: D-glucose 30 mM (n = 8)

- Group D: Insulin 60 mU/L (n = 2)

Insulin 120 mU/L (n = 3)

Insulin 600 mU/L (n = 4)

- Group E: D-glucose 6 mM + insulin 60 mU/L (n = 5)

- Group F: D-glucose 30 mM + insulin 60 mU/L (n = 5)

In the absence of a dose effect of insulin in group D, we performed the last experiments (insulin + glucose i.e. E and F groups) with 60 mU/L insulin concentration. Moreover, in some experiments performed with glucose 30 mM, no difference was observed when insulin concentration was 120 mU/L instead of 60 mU/L (data not shown).

Perfusion lines were saturated by KHB containing insulin at least 30 min before the beginning of perfusion to avoid absorption phenomena during the experiment as reported by others [25].

### NMR methodology

The spectra were obtained using a ^31^P/^13^C double tuned 20 mm probe operating at 9.4T. Liver ATP content was followed by ^31^P NMR and carbohydrate content in natural abundance was assessed by ^13^C NMR. It was therefore possible to calculate the rate of changes of glycogen and ATP content. ^31^P and ^13^C NMR spectra were recorded at 161.9 and 100.6 MHz respectively on a DPX400 spectrometer. The magnetic field was adjusted on the water proton signal. ^31^P NMR spectra were obtained every 2 minutes (240 free induction decays (FID)) without proton decoupling. Radiofrequency pulses (70° flip angle) and 10,000 Hz spectral width were used for data acquisition. ^13^C NMR spectra were proton-decoupled using a gated bilevel mode. ^13^C NMR spectra were obtained every 8 min (500FID) resulting from a 66° radiofrequency pulse repeated every second (25,000 Hz spectral width).

Lorentzian line broadening of 15 Hz was applied before Fourier transformation for both ^31^P and ^13^C NMR spectra. Chemical shift of phosphorylated metabolites was expressed relative to the position of resonance in the frequency scale of an internal reference set (glycerophosphoryl-choline) at 0.47 ppm.^13^C chemical shifts were expressed from an external silicone reference (1.45 ppm). During the initial perfusion period, any liver showing an increase in the intensity of inorganic phosphate resonance occurring with a concomitant decrease in NTP signals probably reflecting some partial lobe ischemia was discarded.

### Analysis

ATP and glycogen levels were estimated from peak areas and expressed as a percentage of the initial value. Relative changes in ATP levels, reflecting the dynamic changes in ATP liver content, were estimated from changes in the area of the spectral peaks of the β-phosphate of nucleoside triphosphates. βATP represents at least 80% of the NTP (around -18 ppm) [27]. The glycogen signal was characterized by a narrow signal at 100.5 ppm (C-1 resonance of glycosyl subunit in glycogen). The difference between the observed change in C1 resonance glycogen represents an estimate for the amount of glycogen that was broken down or synthesized during the time interval. ^13^C NMR quantification of glycogen content at the beginning of KHB perfusion was performed from a calibration curve established with oyster glycogen (0 to 185 mM glycosyl units).

Rate was expressed as %·min^-1 ^or nmol. min^-1^·g^-1 ^and can be calculated from the change in the metabolite signal area, as the linewidth remained constant throughout the experiment:

Rate = 0 : Synthesis = lysis

Rate>0 : Synthesis>lysis

Rate<0 : Synthesis<lysis

Data in the text, table and figures are given as mean ± SEM. Statistical analysis was performed with the software package Excel. A t test was used after a one-way ANOVA to identify significant differences between different perfusate compositions. Probability values of P < 0.05 were considered to be significant.

## Abbreviations

ADP, adenosyl di phosphate; ATP, adenosyl tri phosphate; FID, free induction decay; FFA, free fatty acids; G6P, glucose 6 phosphate; KHB, Krebs-Henseleit buffer; NEFA, non-esterified fatty acids; NMR, nuclear magnetic resonance; NTP, nucleoside tri phosphate; UDPG, uridine diphosphoglucose.

## Competing interests

The author(s) declare that they have no competing interests.

## Authors' contributions

Laurence Baillet-Blanco: contribution to conception and design; acquisition of data; analysis of data and contribution to interpretation of data.

Marie-Christine Beauvieux: contribution to design, acquisition and analysis of data; involved in drafting the manuscript.

Vincent Rigalleau: critical revision.

Henri Gin: contribution to study design; involved in drafting the manuscript.

Jean-Louis Gallis: contribution to study design; analysis of data and large contribution to interpretation of data; involved in drafting the manuscript.
